# Protocol for 3D surface texture modeling and quantitative spectral decomposition analysis in *Drosophila* border cell clusters

**DOI:** 10.1016/j.xpro.2024.103048

**Published:** 2024-07-27

**Authors:** Allison M. Gabbert, Noah P. Mitchell, Emily G. Gemmill, Joseph P. Campanale, James A. Mondo, Denise J. Montell

**Affiliations:** 1 Molecular, Cellular, and Developmental Biology Department, University of California, Santa Barbara, Santa Barbara, CA 93106, USA; 2 Kavli Institute for Theoretical Physics and Department of Physics, University of California, Santa Barbara, Santa Barbara, CA 93106, USA; 3 Biomolecular Science and Engineering Program, University of California, Santa Barbara, Santa Barbara, CA 93106, USA

**Keywords:** Biophysics, Cell Biology, Developmental biology, Microscopy

## Abstract

*Drosophila* border cell clusters model collective cell migration. Airyscan super-resolution microscopy enables fine-scale description of cluster shape and texture. Here we describe how to convert Airyscan images of border cell clusters into 3D models of the surface and detect regions of convex and concave curvature. We use spectral decomposition analysis to compare surface textures across genotypes to determine how genes of interest impact cluster surface geometry. This protocol applies to border cells and could generalize to additional cell types.

For complete details on the use and execution of this protocol, please refer to Gabbert et al.^1^

## Highlights

• *Drosophila* cell-specific transgene expression and ovary dissection and fixation
• Airyscan image acquisition of border cell clusters with high-resolution z stacks
• Extraction of cluster surface to generate 3D models of the surface shape and texture
• Spectral decomposition analysis to quantify differences in surface geometry

## Abstract (editor highlights)

Publisher’s note: Undertaking any experimental protocol requires adherence to local institutional guidelines for laboratory safety and ethics.

## Graphical abstract

**Figure:**
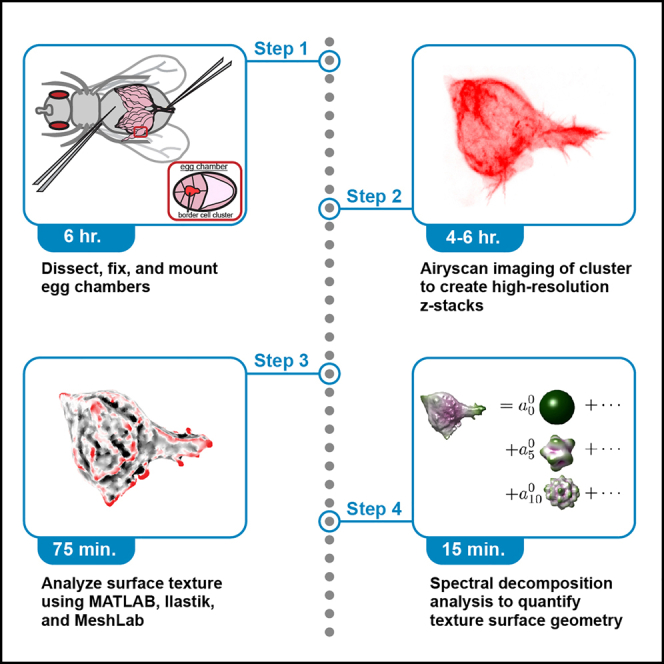

## Before you begin

### Fly genetics and fattening for dissection

**Timing: 4 weeks**

This step involves crossing male and female flies to produce offspring with a genotype of interest for imaging. The female offspring of interest are fattened with yeast powder to increase the size of their ovaries and facilitate ovary dissections. These flies are grown at warmer temperatures to activate Gal4 and induce gene expression.

1. Set up crosses to produce female flies with the genotypes of interest.
  a. Collect five virgin female flies from the desired Gal4 line to cross to each genotype of interest, including a control cross.
    ***Note:*** Include a UAS-transgene encoding a fluorescent membrane marker such as UAS-LifeAct or UAS-PLC***δ***GFP in the border cells to clearly label the surface of the cluster.
  b. Cross five females to two male flies expressing each construct of interest, such as an RNAi or overexpression construct that can be induced in the border cells by the Gal4.
    ***Note:*** Also include an appropriate control construct such as UAS-whiteRNAi.
  c. Grow the crosses at 18°C and transfer the flies to a new vial every 3–4 days.
  d. Adult progeny will emerge after about 20 days.
2. Collect and fatten adult female progeny for dissection.
  a. Collect ∼5 3–5 day old adult female progeny and 1–2 male progeny of each genotype.
  b. Fatten each group by transferring them to fly food vials with the surfaces half-dusted in yeast powder.
  c. Incubate these vials at 29°C for 1–3 days depending on the activation time needed for the Gal4 driver and constructs used.

***Note:*** Three days was sufficient for RNAi knockdown using c306Gal4, but if the construct of interest causes lethality, less time should be used.

**CRITICAL:** The membrane marker should only label the border cell cluster and should not be expressed in the nurse cells.

### Dissection and immunostaining

**Timing: 3 days**

Reagents are prepared and ovaries are dissected from the fattened female flies. The egg chambers are fixed, washed, and immunostained. Finally, the egg chambers are stored in mounting medium.

3. Prepare dissection media and aliquot into 2 mL Eppendorf tubes.
  a. Keep media needed for the current dissections at about 25°C. You will need 150 μL for each dissection.
  b. Store extra media at 4°C.
4. Prepare 0.4% PBST (PBS and Triton X-100) wash buffer.
5. Prepare fresh 4% PFA (paraformaldehyde) diluted with PBS. You will need 500 μL for each genotype.
6. Dissect fly ovaries and collect stage 9 egg chambers.
  a. Dissect ∼5 female flies for each genotype in 150 μL of dissection media.
  b. Remove the ovaries and separate them from other tissues and cuticle.
  c. Pull ovarioles out of the muscle sheath and exclude older egg chambers (stage 12+).^2^
  d. Transfer the egg chambers to 600 μL Eppendorf tubes for each genotype.
7. Fix and stain egg chambers.
  a. After egg chambers have settled in the tube, remove dissection media and add 500 μL of 4% PFA to fix the sample.
  b. Place the tube on a covered rocker for 15 min.
  c. Remove fixative and wash with 500 μL 0.4% PBST wash buffer.
  d. Place on the covered rocker for 15 min.
  e. Repeat this for a total of 3 PBST washes for 15 min each.
  f. Remove wash buffer and add primary antibodies diluted in 200 μL of wash buffer.
  g. Incubate in primary antibodies on a shaker at 4°C for 12–16 h.
  h. The next day, perform 3 washes with the wash buffer for 15 min each on the covered rocker.
  i. Incubate samples in secondary antibodies and any dyes of interest diluted in 200 μL of wash buffer on a covered rocker at 4°C for 12–16 h.
  j. The next day, perform 3 washes with the wash buffer for 15 min each on the covered rocker.
  k. Remove all media from the samples and add ∼60 μL of Vectashield to each tube to use as mounting medium. Store tubes at 4°C.

**Pause point:** Samples can be stored at 4°C to be mounted later. Samples should generally be stored for a maximum of one month.

***Note:*** Dissection, fixation, and staining of each genotype can be done in parallel for efficiency, such as dissecting and fixing the first sample and beginning the second dissection while the first is fixing.

**CRITICAL:** While rocking and incubating, samples should be covered with foil to prevent light exposure.

### Mounting samples for imaging

**Timing: 5 min per sample**

Egg chambers are mounted on a slide with a coverslip. The coverslip is sealed and left to dry.

8. Mount samples on slides.
  a. Take out samples for mounting that will be imaged the following day.
    ***Note:*** Samples can be mounted the same day as imaging, but the mounted samples should be kept at about 25°C for a few hours for the slide to acclimate.
  b. Pipette about 60 μL of sample in Vectashield onto the center of the slide, avoiding slide edges and air bubbles.
  c. Dot each corner of the coverslip with Vaseline to support the coverslip and prevent it from crushing the egg chambers.
  d. Slowly and gently lower the coverslip vaseline side down onto the sample.
  e. Let the Vectashield spread out and seal the sample with thick layers of clear nail polish along the edges of the coverslip.
  f. Add a second coat as a precaution to avoid any sample leakage and dehydration.
  g. Let the slide dry in a flat and dark place such as a drawer at 25°C.
    **Pause point:** Slides can be mounted 1–2 days before imaging, but no longer. Samples should be as fresh as possible for the strongest signal and best quality.
    **CRITICAL:** Mounting right before imaging can cause the slide and sample to warp and move during imaging. Allow at least two hours for samples to dry and equilibrate to the temperature of the room.
    **CRITICAL:** Slides must dry in darkness. Light exposure can degrade sample signal quality.
    **CRITICAL:** Use the coverslip appropriate to the objective/microscope to be used. Our protocol uses a 1.5 coverslip (0.17 mm thickness) for which our objective is appropriately corrected.

### Software and package installation and set-up

**Timing: 1 h**

Software and specific packages and files are installed on the computer in preparation for image analysis. This protocol has been performed using computers with operating systems Windows 10, Windows 11, and OS X versions Yosemite, El Capitan, Sierra, and High Sierra with at least 16 GB RAM, a 6-core processor, and a 1 TB hard drive.

9. Download and install free image analysis software.
  a. ImageJ/FIJI can be downloaded from fiji.sc. Version 1.53 was used. ImageJ is used for initial image processing and to assess image specifications.
  b. Ilastik can be downloaded from ilastik.org. Version 1.4.0rc8 was used. Ilastik is used to classify pixels in the image to generate a 3D model.
  c. MeshLab can be downloaded from meshlab.net. Version 2022.02 was used. MeshLab is used to convert the 3D pixel model to a 3D mesh model of the surface of the object of interest.
10. Download and install MATLAB with additional toolkits.
  a. MATLAB can be downloaded from mathworks.com and is available for free for students and researchers at most universities and research institutions. Version R2020b was used.
  b. Click “Add-Ons” and then search for and download the following toolboxes: Deep Learning Toolbox (version 13.0), Image Processing Toolbox (version 11.0), Statistics and Machine Learning Toolbox (version 11.6), Curve Fitting Toolbox (version 3.5.10), Computer Vision Toolbox (version 9.1), superbar (version 1.5.0.0), and raacampbell/shadedErrorBar (version 1.65.0.4).
11. Download a copy of the ImSAnE and SeptinManuscriptData repositories from https://github.com/idse/imsane and https://github.com/AllisonGabbert/SeptinManuscriptData, respectively.
  a. Do this via one of the options below:
    i. Open Terminal and execute.
      $ git clone --recursivehttps://github.com/AllisonGabbert/SeptinManuscriptData.
      from the parent directory of your choice. This is the recommended option, since in the event of future updates to the codebase, you need only run.
      $ git pull
      on the command line. Note that the - - recursive option above ensures that submodules (which are linked repositories) are included in the local copy.
    ii. Alternatively, navigate to the repository URLs and click Code>Download zip, and then unpack the zipped folders.
  b. Place this directory on the local computer to fit the file organization setup. ImSAnE is used to generate 3D models in the steps: Extract the surface of the cluster using ImSAnE and Generate 3D Curvature Models. The other folders are required for Spectral Decomposition of Border Cell Cluster Shape.
  c. In the ImSAnE folder, open setup.m and run the script “in place” (so that the current working directory is the path to the folder containing setup.m).

## Key resources table

**Table:** 

REAGENT or RESOURCE	SOURCE	IDENTIFIER
**Antibodies**		

Monoclonal rat anti-Ecadherin (1:25 dilution)	Developmental Studies Hybridoma Bank	Cat#DCAD2; RRID:AB_528120
Monoclonal mouse anti-peanut (1:25 dilution)	Developmental Studies Hybridoma Bank	Cat#4C9H4 anti-peanut; RRID:AB_528429

**Chemicals, peptides, and recombinant proteins**		

Hoechst 33342	Sigma-Aldrich	Cat#14533
Phalloidin-Atto 647N	Sigma-Aldrich	Cat#65906
TritonX-100	Sigma-Aldrich	Cat#T8787
Schneider’s Drosophila medium	Gibco	Cat#21720
Phosphate-buffered saline (10x, pH 7.4)	Quality Biological	Cat#119-069-131
Paraformaldehyde, 16% solution	Electron Microscopy Sciences	Cat#15710
VECTASHIELD antifade mounting medium	Vector Laboratories	Cat#H-1000
Fetal bovine serum	Sigma-Aldrich	Cat# F4135

**Deposited data**		

Example data	GitHub	https://github.com/AllisonGabbert/SeptinManuscriptData/tree/main/example_data/plys

**Experimental models: Organisms/strains**		

c306Gal4;UAS-LifeActGFP;Gal80ts	Denise Montell Lab Stock, University of California Santa Barbara	N/A
w[1118]; P{GD8198}v17344	Vienna Drosophila Resource Center	Cat#VDRC_17344
w1118; P{GD11240}v26413	Vienna Drosophila Resource Center	Cat#VDRC_26413
w1118 P{GD1512}v11791	Vienna Drosophila Resource Center	Cat#VDRC 11791
w[∗]; P{w[+mC]=UASp-Septin1.GFP}3	Bloomington Drosophila Stock Center	Cat#BDSC_51346
w[∗]; P{w[+mC]=UASp-Septin2.O}18A/CyO	Bloomington Drosophila Stock Center	Cat#BDSC_91012
c306Gal4, Gal80ts;; UASPLCdeltaGFP/TM3,Ser	Denise Montell Lab Stock, University of California Santa Barbara	N/A

**Software and algorithms**		

ImageJ2 (FIJI) version 1.53	Schindelin et al.^3^	fiji.sc
Adobe Illustrator 2022	Adobe	adobe.com
Prism 9	GraphPad	graphpad.com
Ilastik version 1.3.3	Berg et al.^4^	ilastik.org
MeshLab version 2020.07	Cignoni et al.^5^	meshlab.net
MATLAB R2020b	MathWorks	mathworks.com
ZEN blue version 2.3	Zeiss	zeiss.com/microscopy
GitHub repository	SeptinManuscriptData	https://github.com/AllisonGabbert/SeptinManuscriptData
Zenodo repository	SeptinManuscriptData	Zenodo: https://zenodo.org/doi/10.5281/zenodo.10791061

**Other**		

Halocarbon oil 27	Sigma-Aldrich	Cat# H8773
Eppendorf tube	Eppendorf	Cat# 22363611
Dumont #5 forceps	Fine Science Tools	Cat# 11251-30
Coverslips, size: 40 × 22 mm	Fisher	Cat# 12544-B
Dissecting microscope	N/A	N/A
Zeiss LSM 800 microscope	Zeiss	LSM 800
Eisco concave microscope slides	Fisher Scientific	Cat#S99369

## Materials and equipment

• Dissection media: Add 40 mL of Schneider’s *Drosophila* medium to 10 mL of Fetal bovine serum (FBS) and adjust pH to 6.9-7.0. Final dissection media is 20% FBS and 80% Schneider’s medium.

Store at 4°C.

***Alternatives:*** For fixed imaging, flies can be dissected in PBS instead of dissection media without adverse effects.

• 0.4% PBST wash buffer:

**Table:** 

Reagent	Final concentration	Amount
10X PBS	1X	10 mL
Triton X-100	0.4%	400 μL
Milli-Q water	N/A	90 mL

***Note:*** Stir with a magnetic stir rod to dissolve Triton X-100 before use.

Store at 25°C for 2 weeks.

• 4% Paraformaldehyde (PFA): Dilute PFA stock with 1X PBS. Only prepare the volume needed, with 500 μL used per sample.
Keep at 25°C but use within the day. Opened PFA stocks can be kept sealed at 4°C.

**CRITICAL:** PFA is a fixative and health hazard. Read the accompanying safety data sheet and avoid inhalation and contact with the skin and eyes. Wear proper PPE including gloves and a lab coat and work in a fume hood.

## Step-by-step method details

### Airyscan image acquisition

**Timing: 4–6 h**

Border cell surface topologies are determined after super-resolution imaging. Border cell clusters are imaged using an inverted Zeiss LSM800 scanning confocal microscope running Zen Blue, version 2.3, and outfitted with a circular arrayed 32-channel Airyscan 1 detector. Images are denoised by Airyscan processing using the Zen software. The resulting images are at an XY/Z resolution of 120 nm × 120 nm which allows for the fine-scale surface reconstruction of border cell membrane/cortex. Here we describe the imaging of GFP-tagged LifeAct or pleckstrin homology (PH) domain in fixed samples. Reconstruction of other tags or fluorophores is possible after confirming they can be imaged by Airyscan and produce images with sufficient resolution and noise reduction.

1. Load slide onto confocal microscope universal slide holder.

***Note:*** The coverslip will be face down and closest to the objective on an inverted microscope.

***Note:*** Airyscan imaging is prone to blur if the sample moves. Ensure the microscope is mounted to a properly functioning anti-vibration table and the slide is snug in the sample holder.

**Figure 1:**
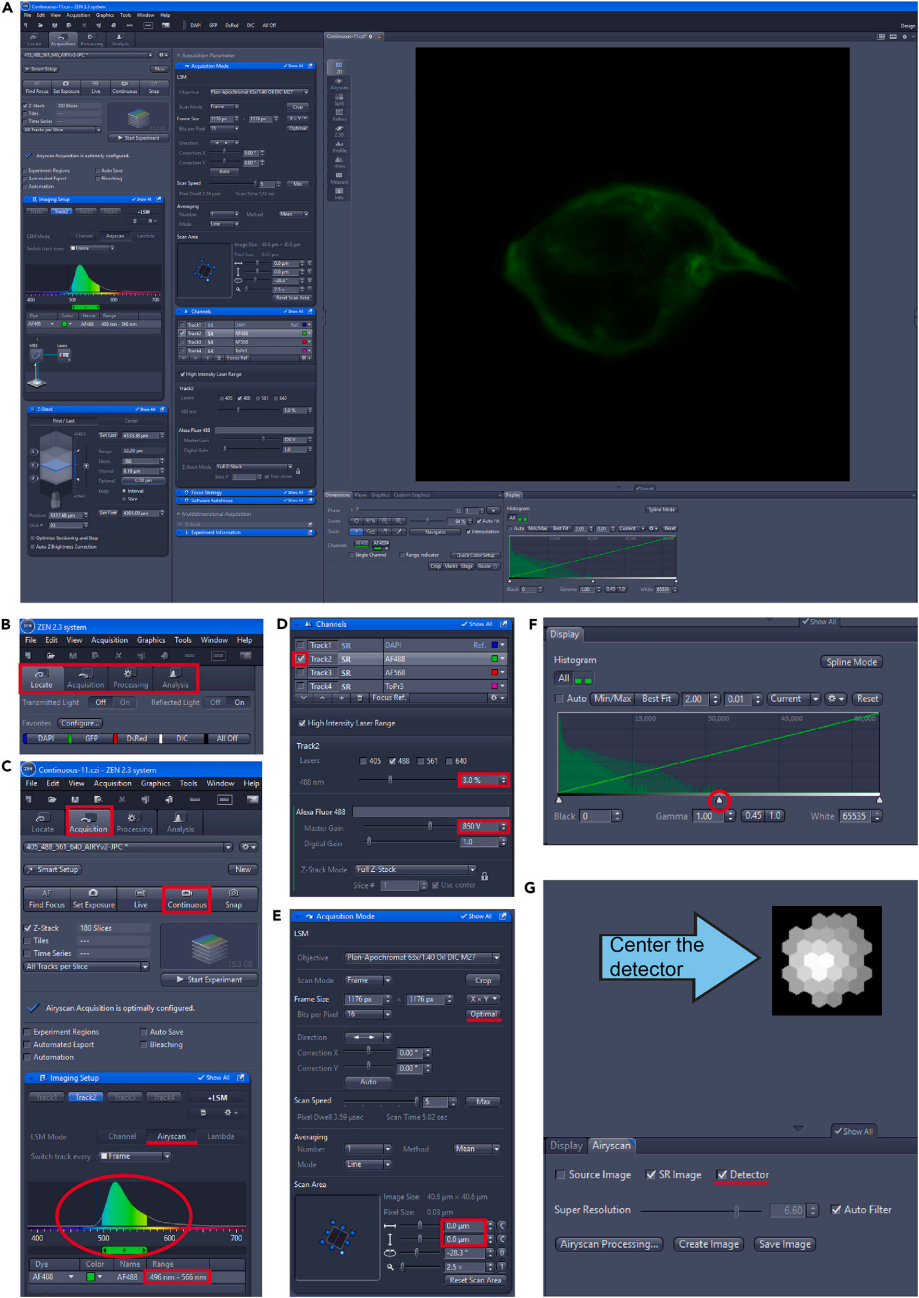
Acquire Airyscan images through Zen (A) Full screen view of Zen during Airyscan imaging setup. (B) Zen opens with a view of the Locate tab. (C) Switching to the Acquisition tab enables changes to the image acquisition settings. The LSM Mode is switched to Airyscan and the imaging range matches the fluorophore of interest. A range of about 496–566 nm clearly captures GFP signal. (D) View of the Channels window. Only the track(s) of interest is selected. Laser Power and Master Gain are adjusted for imaging. (E) View of the Acquisition Mode window. Frame size is decreased during initial set up but is increased to Optimal settings during sample image acquisition. Scan area can be adjusted. (F) Histogram of signal range at current imaging settings. The histogram covers about half of the total range, illustrating optimal imaging settings. (G) View of the Airyscan Detector. Checking the “Detector” box shows the detector. This illustrates if the detector is properly calibrated or if it requires alignment.

***Note:*** Select egg chambers free from damage and with migrating border cell clusters.

***Note:*** Multiple positions can be marked using the tiles module in Zen if using a motorized XY-stage. This allows one-click movements between positions that were specified at lower magnification while at higher magnification (next step).

2. Open the Zen Blue software.
3. Select the 10x/ 0.3NA EC-Plan Neofluar objective from the Zen software or the touch screen.
4. Use the Locate tab (Figures 1A and 1B) in the Zen software to select the GFP filter and press “on” to open the shutter.
  a. Locate and obtain focus on a single stage 9 egg chamber in the center of the field of view.

5. Apply a single drop of Immersol 518F immersion oil to a 63x/1.4NA PlanApochromat immersion objective.
  a. Return to the marked position and center the field of view on a single border cell cluster.

***Note:*** Do not allow the objective to touch the slide without a layer of immersion oil.

***Note:*** Use the fine focus knob (inner dial) for immersion objectives to prevent them from pushing on the coverslip and smashing/damaging the egg chambers.

***Note:*** Once oil is used, do not return to any air objective.

6. Click the Acquisition tab (Figures 1A–1C).
  a. Select the checkbox for z-stacks (Figure 1).
  b. Select the Airyscan imaging mode (Figure 1C).
  c. Activate a single track with the 488 nm excitation wavelength (Figure 1D) and collect the emission spectra from 496 to 566 nm (Figure 1C).

***Note:*** Multiple tracks can be acquired. Surface reconstructions require fluorescent LifeAct or PH-domain markers for analysis. If multiple markers stained with different fluorophores are acquired, ensure each fluorophore is imaged on a separate track (Figure 1D).

7. Determine the Airyscan laser power and gain setting for image acquisition settings at low XY resolution.
  a. Set the XY frame size to 256 × 256 pixels with 16 bits/pixel, bi-directional imaging, and the fastest scan speed (Figure 1E).
  b. Selecting only the 488 nm track, press the “Continuous” button to view the border cell cluster.
  c. Center the cluster using the motorized joystick. Zoom the scan area to 2.5× zoom and rotate so that the anterior/posterior is represented by the left and right sides of the image respectively (Figure 1E).
    ***Note:*** Do not adjust the scan area vertically or horizontally. Keep the imaging area in the center of the scan area.
  d. Adjust the laser power and gain for optimal signal-to-noise pixel intensities.
  e. Set the gain between 750-850 V and adjust the laser power between 1%–5% (Figure 1D).
    ***Note:*** Aim for a histogram that limits noise (left side of histogram) and has signal that reaches 50% of the detection limit of the Airyscan detector (Figure 1F, signal spans about half of the x-axis).
    **CRITICAL:** Signal-to-noise is crucial. Limit pixel saturation or using gains beyond 850 volts. Minimize overexposure by using the range indicator button and ensuring no red pixels are present or that there are not a significant number of positive pixels present as background noise.
    ***Note:*** If multiple tracks are imaged by Airyscan, set laser power and gain for each track independently.
8. Align the Airyscan detector while using the “Continuous” imaging mode.
  a. While in “Continuous” mode (Figure 1C), select the “Airyscan” tab to the left of the image and switch from “Display” to “Airyscan” in the tabs below the image (Figure 1G).
  b. Check the “Detector” box to activate a display of the circular Airyscan detector (Figure 1G).
  c. Inspect the detector display. If properly aligned, the center hexagon will be the brightest white color with a gradient of light to dark gray moving to the edges of the detector array.
  d. Choose the first and last z-slice using the first and last buttons in the z-stack window while using the fine focus knob.
    ***Note:*** The Airyscan detector performs self-alignment when the histograms are sufficiently bright and the histogram covers 50% of the x-axis of the display. In rare instances, the Airyscan detector needs alignment. This happens if this gradient is not present or not centered on the detector. This should be used rarely if at all as the detector constantly aligns itself during imaging.
    i. In the top toolbar, select System Maintenance and Calibration, and continue.
    ii. Select “Airyscan detector adjustment”.
    iii. Manually slide the detector’s x and y position or check the automatic adjustment box to allow the detector to automatically adjust its position while in “Continuous” mode. Uncheck this box when calibrated.
9. Image with Airyscan near super-resolution.
  a. Select optimal z-slice interval and optimal XY resolution (Figure 1E).
    ***Note:*** Acquire images at the fastest imaging speed and without averaging at 16-bits.
  b. A blue checkmark will appear to indicate “Airyscan acquisition is optimally configured” (Figure 1A). If not, confirm that all settings are optimal.
  c. Select the “Start Experiment” button to run the imaging experiment. This will take between 1-3 h depending on the number of z-stacks.
10. Process Airyscan image file in Zen.
  a. With the raw Airyscan image open, select the processing tab next to the acquisition tab (Figure 1A). Select Airyscan Processing, check 3D processing, then press “Apply”.
    ***Note:*** This step uses a lot of RAM. The progress bar is located at the bottom of the screen.
  b. Save the Airyscan processed file.
11. Optional: Image additional position(s).
  a. Select a border cell cluster at a different saved position in the Tiles module and adjust laser power and gain if needed. Follow the protocol from Step 4.

**CRITICAL:** Once you switch to the 63× oil objective you cannot return to lower objectives. Do not move up until you have finished finding positions and imaging at the lower objectives.

**CRITICAL:** After imaging a slide with the 63x oil objective, the slide will be covered in oil. This slide cannot be viewed with an air or water objective any longer, so it is difficult and not recommended to reuse slides for multiple imaging sessions. Therefore, allocate plenty of time to perform Airyscan imaging so the samples will not be wasted.

**CRITICAL:** Capture a few z-slices above and below the cluster so the cluster is not cut off at either end. If the z-slice starts or stops within the cluster, the model built in the next step will have a flat and unquantifiable surface.

### Extract the surface of the cluster using ImSAnE

**Timing: 1 h**

This section converts an Airyscan image file to a 3D point cloud model of the cluster surface in preparation for 3D model surface construction and analysis.

**Figure 2:**
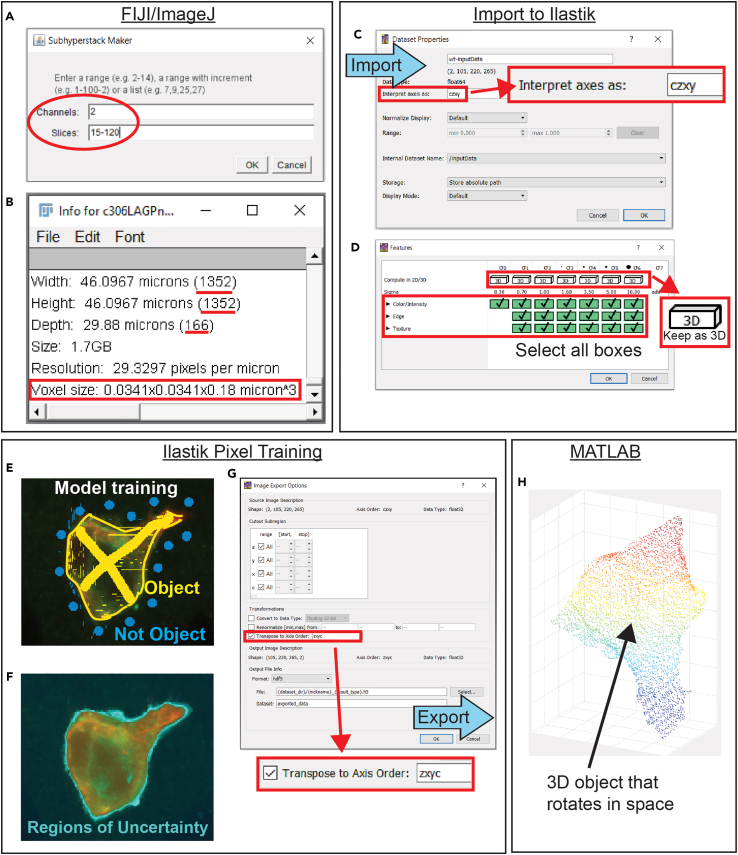
Map the surface of the cluster using ImSAnE (A) Subhyperstack maker in FIJI. This window allows for the selection of specific channels and slices of interest to include in the analysis. (B) Show Info window in FIJI. This window provides a list of image specifications and details associated with the image including dimensions and voxel size. (C) The Dataset Properties window in Ilastik allows for the adjustment of properties when importing compressed h5 files. Ilastik interprets axes in a different order than the other softwares utilized in this protocol, so the axes order must be transposed during data import and export with Ilastik. (D) Features selection window in Ilastik. The choice of features corresponds to what area and what characteristics the pixel classification software will consider during training. It is recommended to include all areas and characteristics for accurate results. As the data is in 3 dimensions, this training is performed in 3D. (E) Training pixel classification software in Ilastik. The yellow labels signify pixels to classify as part of the object of interest and the blue labels signify pixels to exclude. This labeling should be performed on a range of z-slices and should help clarify ambiguous areas. (F) Running “Live Update” in Ilastik after preliminary label markups shows a predicted segmentation of the object based on the current labeling information. Viewing regions of uncertainty in live mode can highlight regions that require further labeling. (G) Image Export Options window in IIlastik. Similar to importing data into Ilastik, exporting data from Ilastik also requires transposing of the axis order for downstream analysis in additional softwares. For use in MATLAB, the order is switched back to “zxyc” before export. (H) 3D point cloud model in MATLAB. The data exported from Ilastik and imported back into MATLAB is represented as a 3D point cloud. This model should clearly illustrate the 3D surface of the cluster without additional objects present or disruptions of the surface such as flat regions or inclusion of background areas. This model can rotate in space.
  d. Save this new substack file as a TIF, saved or copied to the working directory used for later analysis.

***Note:*** Be conscientious of file names such as this TIF, as names will be referenced throughout the following analysis script as new files are created.

12. Convert multi-channel czi file to one channel TIF file in ImageJ/FIJI.
  a. Open the Airyscan image czi file in ImageJ/FIJI as a hyperstack.
  b. Scroll through the z-slices and choose a range of z-slices that will capture the entire cluster from top to bottom without cutting off either end. This may be the entire range imaged or a subset of the imaged slices.
  c. Make a substack of the original image by opening the “Image” menu in the toolbar and selecting “Stacks”, “Tools”, and “Make Substack…”. If the image has more than one channel, choose the channel of the cell membrane marker. Enter this chosen channel and z-slice range to use for this analysis (Figure 2A).

13. Note the size and resolution of the TIF file in ImageJ/FIJI.
  a. In ImageJ/FIJI, select the TIF image, open the “Image” menu in the toolbar, and select “Show Info…”.
  b. In the new text window, “Width”, “Height”, and “Depth” are shown in microns and pixels (the values in parentheses) (Figure 2B). This is often near the very top or bottom of the text window. Note the pixel values as the dimensions of your image.
    i. This is also denoted by the terms SizeX, SizeY, and SizeZ.
  c. A few lines below this, “Voxel size” is presented in the format of AxBxC micronˆ3 (Figure 2B). These values represent the width, height, and depth of each voxel. Note these three values.
14. Customize the modeling script to import specific TIF.
  a. Open the tissue cartography modeling script STAR_Methods_Tissue_Cartography_Modeling.m in MATLAB for editing.
  b. Follow the directions in the comments to replace the placeholder “MYFILENAME” with the name of the TIF (line 26) and the desired output file name (line 82).
  c. Adjust the number of channels if needed (default is one channel for the membrane).
  d. Input image size and resolution values into presented matrices.
  e. Close ImageJ/FIJI.
15. Import data, generate an h5 file, and upload it to Ilastik.
  a. Run the tissue cartography modeling script until the first “pause”. This should import the TIF data, rescale it, and compress it into an h5 file.
    ***Note:*** Surface detection is done by the Ilastik Detector in ImSAnE (/imsane/+surfaceDetection/IlastikDetector.m). Detection parameters are passed to the Ilastik Detector by myDetectOpts in TissueCartographyModelingDevCell.m. These options will need to be changed depending on biological question, image size/resolution, and compute resources available. For example, higher values of “ssfactor” (sub-sampling factor) allow for faster processing and less intensive computation, but a loss of resolution and potentially of finer cellular structures. “sigma” controls the amount of Gaussian smoothing applied to the image, “rmRadialOutliers” controls removal of radial outliers (poorly or non-connected surfaces), and “dildisc” controls the size of a dilation disc that smooths over possible holes in a surface (for example due to staining cortical actin instead of cell membrane more directly). We provide values appropriate to surface detection of border cells visualized with LifeActGFP for quantification of membrane curvature under our imaging conditions, but this will require optimization for different image types or biological questions (for example fine filopodia detection will likely require lower values for sigma and dildisc). Note that meshes are output with resolution based on the subsampled data.
  b. Open Ilastik and under “Create New Project”, select “Pixel Classification”. Save this in the working directory.
  c. In the first “Input Data” tab, there is an empty table. In the “Raw Data” tab of the table, click the button “Add New…” and choose “Add separate Image(s)...”.
  d. Select the h5 file to import it.
  e. Double-click the “Axes” column to transpose the axes. A text window will appear that reads “Interpret axes as:”. In the box, type “czxy” and click “OK” (Figure 2C).
    ***Note:*** Image acquisition settings may vary, so it is possible an imported image does not require axes transposing, or a different axes order may be required. Ensure that the dimensions and visualization of the x, y, and z axes in Ilastik match the original image.
  f. Click on the second tab on the left called “2. Feature Selection”. Click the button “Select Features…” and highlight or check all of the boxes for “Color/Intensity”, “Edge”, and “Texture” (Figure 2D). Hit “OK”.
16. Train the Pixel Classification tool in Ilastik.
  a. Click the third tab called “3. Training”. The yellow “Label 1” will label one group of pixels (which ImSAnE will consider foreground) and the blue “Label 2” will label a second group of pixels (which ImSAnE will consider background).
  b. Click the brush icon and Label 1 or Label 2 to draw on and label regions of the image in the x, y, and z-axis perspective (Figure 2E).
  c. Adjust brush size with the “Size:” dropdown selection and erase the brush with the eraser tool if needed.
  d. Scroll up and down in each window to adjust which slice is in view.
  e. To increase image size, click on the upward triangle “Zoom to fit” icon on each image.
  f. After a preliminary mark-up, select “Live Update” and wait for Ilastik to update.
  g. Click on eye icons to change viewing modes while in Live Update mode and scroll through slices.
  h. If predictions for Labels 1 and 2 are not accurate, additional markups are needed for further learning.
  i. Uncertainty can highlight regions where Ilastik is less confident which require further labeling (Figure 2F).
  j. After completing Pixel Classification training, save the prediction by saving the project.

**CRITICAL:** Turn off “Live Update” before adding additional markups to images. Labeling images while running Live Update can cause the software to crash if run on a workstation with insufficient RAM. Turn Live Update back on after adding labels and give the software time to process the new markups.

**CRITICAL:** To prevent loss of work, save the project often in case Ilastik crashes.

**CRITICAL:** The quality of the final image depends on training the Ilastik model well. If there is substantial uncertainty on the edges, then the output will just be a blob without fine texture. Careful training and taking the time to train on small details on the edges will result in a better 3D model that captures the finer details.

**CRITICAL:** Avoid over-training the data as this both slows down Ilastik and reduces the classification generalizability to other clusters. Train the model just enough to accurately classify the cluster and iterate through the cluster, adding additional markups to areas with high uncertainty, rather than overtraining the model from the start.

***Note:*** For larger sample sizes, rather than manually training Ilastik for each image, Ilastik can batch-process files after an initial round of training. To do this, import a small representative data set of h5 files and train them all, being careful to validate the training to ensure classification accuracy. Switch between samples by selecting the image under “Current View”. After training, perform prediction export as detailed below. To perform batch processing, click “Select Raw Data Files” and then import your data set. Click “Process all files”. The prediction files should then be output in their original folder as h5 files.

17. Export prediction from Ilastik.
  a. Click the fourth tab called “4. Prediction Export”. The source should be “Probabilities”.
  b. Click the button “Choose Export Image Settings…” and check “Transpose to Axis Order:” (Figure 2G). In the box enter “zxyc” and click “OK”.
  c. Click the button “Export” or “Export All” (both will work).
  d. Save the project and close Ilastik.

**CRITICAL:** The resulting probabilities field – and therefore also the resulting surface mesh – depends on your pixel classification. Be sure to have uniform standards across experimental conditions for assigning pixel labels. Better yet, use a single Pixel Classification Project that has been sparsely trained on disparate data and use the Batch Processing feature to apply the classification to all other data across conditions.

**CRITICAL:** Ensure the output h5 files are named as “<filename>_Probabilities.h5”, as indicated in the export preferences so that the script can then find the correct files to load.

### Generate 3D Curvature Models

**Timing: 15 min**

This section converts the h5 probabilities file into a point cloud using the ImSAnE script. Then the script converts the point cloud to a cleaned surface mesh in MeshLab for reimportation into MATLAB to create a 3D curvature model with heatmap coloring.

18. Convert probabilities into point cloud OBJ file in MATLAB script.
  a. Ensure your Ilastik probabilities file is in the same directory as the MATLAB script STAR_Methods_Tissue_Cartography_Modeling.m.
  b. Follow the directions in the comments to replace the placeholder “MYFILENAME” in lines 116 and 120 with the desired name for the OBJ file.
  c. Resume the MATLAB script by pressing any key in the command window. This will run the ImSAnE script until the second “pause”.

***Note:*** This will import the h5 probabilities file, detect the surface, and create a 3D point cloud (Figure 2H). This will be saved as an OBJ file.

**Figure 3:**
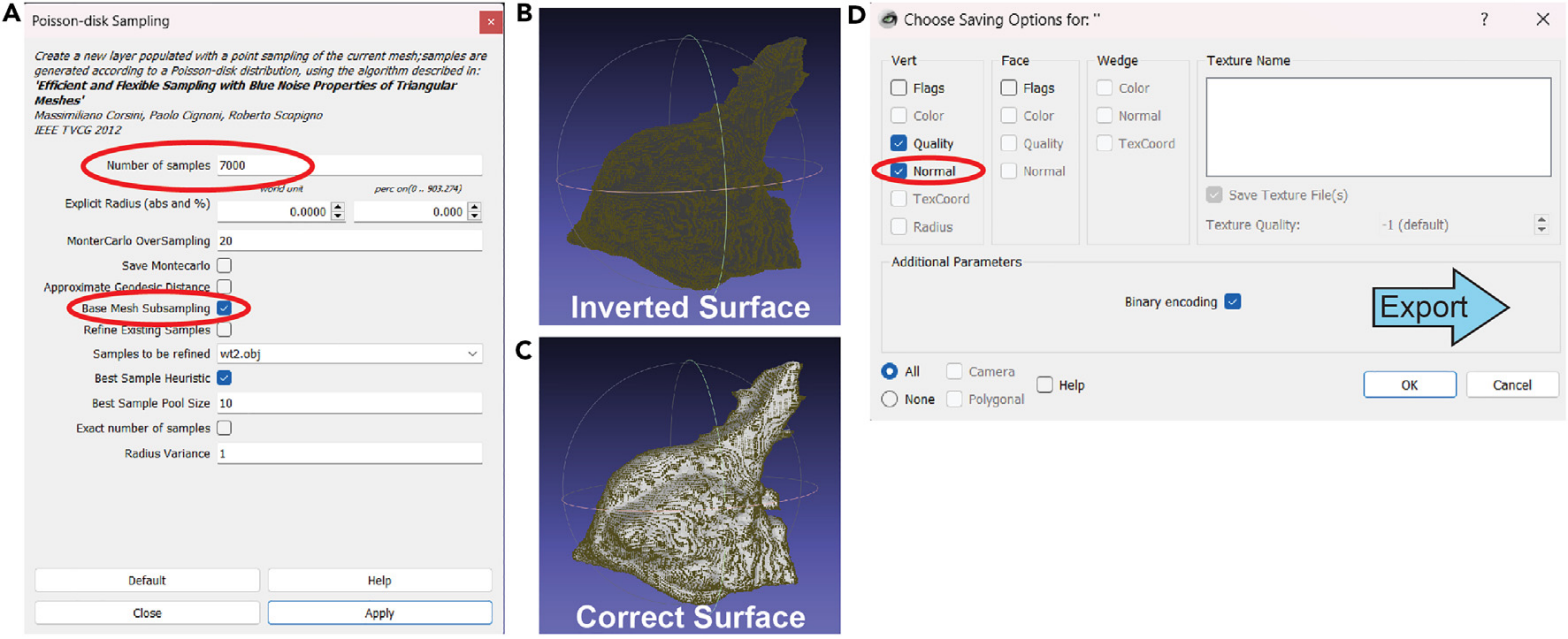
Generate 3D meshes in MeshLab (A) Poisson-disk Sampling window in MeshLab. After importing the h5 file with the point cloud from MATLAB, Poisson-disk sampling is performed on the mesh. The number of samples should be chosen with attention to the complexity of the surface; more complex surfaces should have higher sample numbers (∼10000), while smoother or rounder surfaces should have lower sample numbers (∼5000). Starting at 7000 is generally an efficient choice. Base Mesh Subsampling should be selected and then click “Apply”. (B) If the resulting mesh following surface reconstruction is dark, this means that the surface is inverted from the actual cluster. This is resolved by inverting the face mesh. (C) The surface should appear light if the border cell cluster surface has the correct side facing outward. (D) After verifying that the surface is not inverted, then the file is exported. In the Saving Options window in MeshLab, select ‘Normal’ under ‘Vert’, then click ‘OK’.
  d. Under “Filters”, select “Normals, Curvatures, and Orientation”, then click “Compute Normals for Point Sets”.
  e. Click “Apply” and then “Close”.
  f. Under “Filters”, select “Remeshing, Simplification, and Reconstruction”, then click “Surface Recon: Screened Poisson”.
  g. Click “Apply” and then “Close”.
  h. If the surface is inverted (appears mostly dark green) (Figure 3B), under “Filters”, select “Normals, Curvatures, and Orientation” and then click “Invert Faces Orientation”. If the figure appears correct (Figure 3C), do not invert the faces.
    ***Note:*** It may be helpful to drag the object around to rotate it in MeshLab. This allows you to see the surface better and verify that the object has been correctly meshed and that your z-stacks were not cut off during imaging.
  i. Export the mesh by clicking “File”, then “Export mesh as…”. Check the box for “Normal” under “Vert” (Figure 3D).
  j. Click “OK” and then verify that there is a PLY file in your working directory.
20. Create a 3D curvature model in MATLAB using surface mesh.
  a. Return to the MATLAB script STAR_Methods_Tissue_Cartography_Modeling.m and follow the directions in the comments to replace the placeholder “MYFILENAME” with the PLY file name in line 132.
  b. Resume the MATLAB script by pressing any key in the command window. This will run the ImSAnE script until the third “pause”.
  c. The script will then prompt you to change the file name in line 308 for your output file name.
    ***Note:*** This step may take a couple of minutes and you will see lines being output in the command window.
  d. Complete the MATLAB script by pressing any key in the command window. The script will finish and there should be a curvature file in your directory.

***Note:*** It is best practice to save a copy of the script with the specifications for each image that you analyze. Do this by pasting the following line in the command window and inputting your custom file name in place of MYFILENAME:

19. Translate the point cloud into a surface mesh in MeshLab.
  a. After the MATLAB script pauses, open MeshLab.
  b. Under “File”, select “Import Mesh…”, and then select the OBJ file that was exported from MATLAB. An object should now appear in MeshLab.
  c. Under “Filters”, select “Sampling” and then click “Poisson-disk Sampling” (Figure 3A).
    i. In the pop-up window, there will be an option to select the number of samples (Figure 3A). 7000 is a good general choice.
      ***Note:*** The choice for sampling depends on the image resolution. In general, around 5000 works for simple shapes, and 10000 works for complex shapes. For most border cell clusters, 7000 is a good choice. If sampling errors appear, this value can be adjusted.
    ii. Check “Base Mesh Subsampling”.
    iii. Click “Apply” and then “Close”.
    iv. The bottom right corner should display “Applied filter Poisson-Disk Sampling”.

>copyfile(“STAR_Methods_Tissue_Cartography_Modeling.m”, “MYFILENAME.m”)

### Spectral Decomposition of Border Cell Cluster Shape

**Timing: 15 min**

Having obtained a surface model of the border cell cluster as a triangulated mesh, we now compute the degree of surface roughness at varying spatial scales. At the scale of the whole border cell cluster, this surface may protrude or pucker in some direction(s) relative to others. At the same time, at small spatial scales, the surface may be corrugated or smooth in texture, depending on the structure and mechanics of the interactions between the plasma membrane and the cytoskeleton or other molecular components. Separating the surface topography by its variations across different spatial scales leads to insights into how septins influence border cell cluster morphology – both via large-scale actin-rich protrusions and via small-scale membrane ruffles. Here, we quantify surface geometry using spherical harmonics and mean curvature flow, in an approach adapted from tools presented in Mitchell & Cislo.^6^

21. Open script to analyze surfaces.
  a. To analyze surfaces open the basic spectral analysis script script_spectralAnalysis_basic.m in MATLAB, either by double-clicking the file from a Finder window or running the line below in MATLAB:
    >cd /path/to/my/repo/SeptinManuscriptData
    >cd spectralAnalysis
    >edit script_spectralAnalysis_basic.m
    ***Note:*** /path/to/my/repo/ should be replaced by the actual path to where the SeptinManuscriptData repository was cloned.
  b. Edit the path datadir in line 33 of the script to match the path to the surface data of interest on the local computer.
22. Run the script_spectralAnalysis_basic.m script, which runs sequentially over each surface PLY file stored in the directory datadir.
  a. First we map the triangulated mesh saved in the PLY to a sphere.
    ***Note:*** This is done using the method of Kazhdan et al.^7^ implemented in the function conformalized_mean_curvature_flow(). This map enables the user to analyze other kinds of metrics, such as Euclidean displacement of each vertex from its conformally mapped position on the sphere, but here we simply use the difference between the radial position and the radius of the appropriately scaled sphere. To make this measurement, having a unique and natural mapping to the sphere is important since we use vertex positions of the mapped, spherical mesh to define the patterns of spherical harmonics on the specific mesh in the next step. We measure the amount that the mesh protrudes from this reference sphere as $\delta r=|r-r_{0}|-R$ , where r is the position of a given vertex of the mesh in 3D space, $r_{0}$ is the center of the spherical mesh to which the 3D mesh is mapped, $|r-r_{0}|$ denotes the Euclidean distance between the two locations, and R denotes the radius of the spherical surface to which the mesh is mapped.
  b. We then compute the eigenvectors of the Laplace-Beltrami operator defined on the mapped, spherical surface mesh. This gives us a measure of participation of each mesh vertex in each spherical harmonic.
    ***Note:*** Each spectral mode $Y_{l}^{m}\left(\theta ,\varphi \right)$ is specified by two indices (l,m). The spectral power for each mode $Y_{l}^{m}\left(\theta ,\varphi \right)$ is the amount of weight given to a spherical deformation described by the pattern of that mode with unit norm, such that $\delta r=\Sigma_{l=0}^{\infty }\Sigma_{m=-l}^{l}{{{a^{m}}_{l}Y}^{m}}_{l}$.
  c. We then bundle different spherical harmonics with the same value of l.
    ***Note:*** Each value of l defines a spatial scale of variation whose topographic features we query. We chose to simply add the absolute values of each component $a_{l}^{m}$ for a single measure of weight at each value of l: $A_{l}=\Sigma_{m=-l}^{l}\left|a_{l}^{m}\right|$.
  d. Lastly, we measure the statistics of the power spectrum across different samples included in the analysis.
23. If there are multiple conditions (e.g., WT, knockdown, and over-expression), compare spectral analysis results across these conditions by running a script to analyze and compare all conditions at once.
  a. A template is given in script_spectralAnalysis_acrossConditions.m, found in the spectralAnalysis directory within the SeptinManuscriptData repository.
  b. Open this file in MATLAB for editing.
  c. Put all PLYs from each condition into a folder and have these folders all lie within the same parent directory.
  d. Modify line 27 to list the directory names of the conditions to compare.
    ***Note:*** For example, to compare PLYs within directories named ‘knockdown’, ‘wildtype’, and ‘overexpression’, write
    >dirs = {‘knockdown’, ‘wildtype’, ‘overexpression’} ;
  e. Modify line 31 to name these conditions on any plots that are made.
    ***Note:*** Short names are helpful to keep the plot readable. For example, we can describe the conditions reflected in the directories used before:
    >shorthand = {‘KD’, ‘WT’, ‘OE’} ;
  f. Navigate to the parent directory where the subdirectories for each condition are stored, either by clicking through the MATLAB file browser or by executing
    >cd /path/to/my/data
    in the Command Window, where /path/to/my/data should be replaced with the true file path.
  g. Run the code from that location.
24. Interpret the results.
  a. Plots will be saved to disk showing the spectral weight as a function of the index l.
  b. In these plots, we have summed over all indices m=−l,−l+1,...,l for each l, which defines a characteristic spatial scale of variations in δr over the surface of the reference sphere.
    ***Note:*** Heuristically, the index l provides a spatial frequency of the pattern of deformation required to transform a sphere to the mesh surface, while m can be viewed as indexing the angular orientation of that pattern. Larger weight in low l modes denote the presence of large-scale variations in radial displacement (protrusions), whereas larger weight in high l modes denote the presence of fine-scale variations in radius (surface roughness).
  c. We compared the spectral weight of l=1 modes to capture the amount of polarized protrusion across genotypes.
  d. We also compared high l modes (we chose an arbitrary cutoff of l>6) to relate the amount of small-scale surface roughness between conditions.

***Note:*** We also ignore modes reflecting roughness on a scale that is comparable to (or smaller than) the sampling resolution of our meshes (i.e., smaller than the typical size of a triangle in the mesh triangulations).

***Note:*** If a warning message box appears saying “File …spectralAnalysis.m is not found in the current folder or on the MATLAB path,” then select “Add to Path” rather than “Change Folder.”

***Note:*** Each mesh includes a list of vertex positions in 3D space, and these positions have units of (subsampled) pixels. We convert the positions to microns in the script. If one mesh was generated from a different resolution data volume than others, indicate that resolution in microns per (subsampled) pixel in a txt file with the name <mesh filename>_resolution.txt. A valid resolution text file should have been output along with the PLY file in the previously run ImSAnE script.

***Note:*** In the interpretation, the weight of each mode (l,m) is the amount of deformation amplifying a mode with unit norm. That is, when we compute the eigenfunctions of the spherical mesh

**Figure 4:**
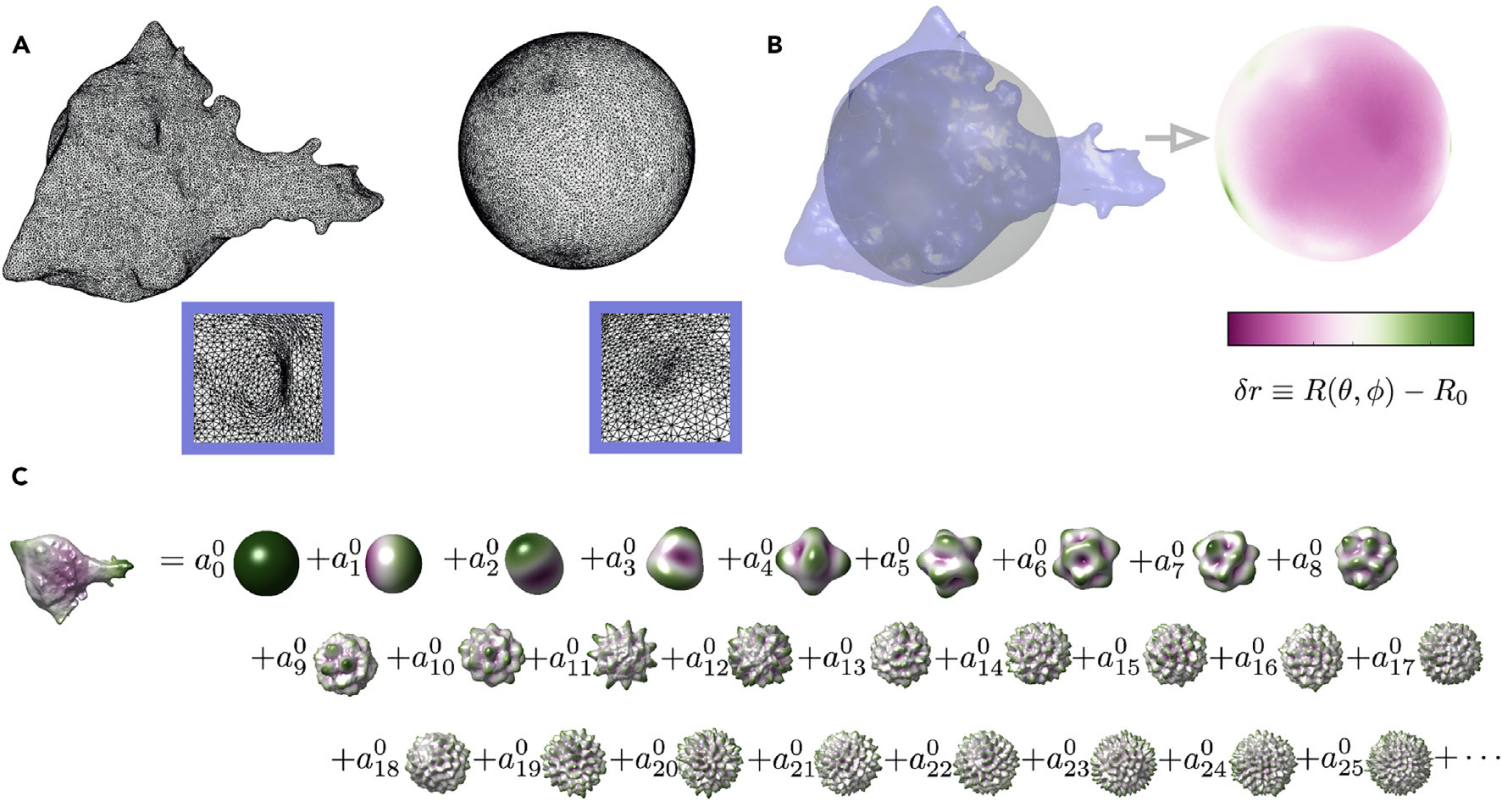
Spectral decomposition of border cell cluster shape Decomposing the surface into spherical harmonics provides a quantitative measure of shape. (A and B) Mapping each mesh to a sphere using conformalized mean curvature flow provides a measure of protrusion from a reference surface. (A) The mesh triangulation of the border cell cluster surface acquired earlier maps to a sphere in a manner that preserves angles of the triangulation — a ‘conformal’ map. (B) Subtracting each mesh vertex’s radial coordinate R(θ,φ) from the radius of the sphere $R_{0}$ provides a measure of radial distance δr. Note that radial distance measurement is patterned on the sphere using the mapped configuration’s spherical coordinates (θ,φ), so that δr gives the radial displacement that each vertex acquires while mapping the spherical mesh to the true surface geometry. (C) We then decompose this signed distance field on the sphere into components of increasingly fine spatial scale using the spherical harmonics as a set of basis functions. The coefficients $a_{l}^{m}$ provide a measure of spectral weight for each pattern of deformation from a spherical state. For clarity, we show only one pattern (m=0) for each index l.

> laplaceBeltrami = cotmatrix(Urescaled - sphereCenter, mesh.f) ;

> [V,∼] = eigs(laplaceBeltrami,nModes,0);

(Figure 4C), we see that the norm of each mode is 1:

> vecnorm(V’, 2, 2) == 1

The spectral weight of each mode amplifies that mode’s deformation to match the amount of that pattern measured in the mesh relative to a reference sphere obtained by conformalized mean curvature flow (Figure 4A).

## Expected outcomes

Proteins such as septins can regulate border cell surface geometry by reshaping cell membranes.^1^ In this protocol, we alter gene expression in the border cells and assess their effects on border cell surface texture. Imaging a border cell cluster through Airyscan produces a high-resolution z-stack czi file. Following segmentation with Ilastik, MATLAB generates a 3D point cloud model of the cluster. The extracted surface is further modeled as a 3D mesh in MeshLab software. This mesh is customized in MATLAB to color-code surface regions of concavity and convexity. A collection of meshes from multiple genotypes or categories can be quantitatively analyzed through spectral decomposition analysis in MATLAB. The spectral analysis script provides a readout of spectral weights across categories through a low-order mode comparison and a high-order mode comparison which can be interpreted to determine the relative complexity of a surface.

**Figure 5:**
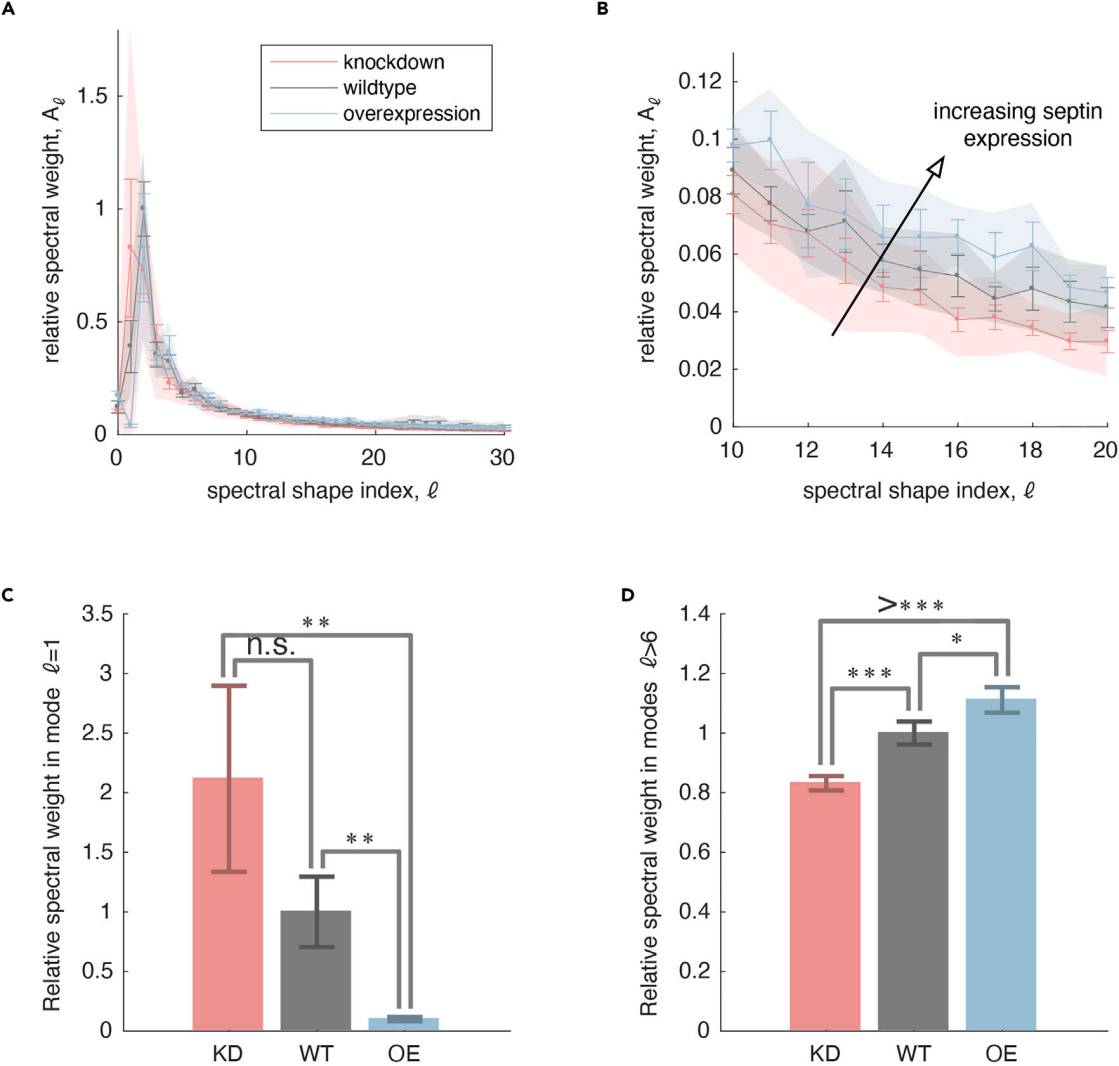
Expected outcomes Comparing shape spectra across conditions reveals the effect of septin perturbations on border cell cluster shape at different spatial scales. (A) Our analysis yields a measure of spectral weight. We chose to add the weights indexed by each index m, which ranges between −l<m<l for each index l, so that $A_{l}=\Sigma_{m=-l}^{l}\left|a_{l}^{m}\right|$. For different conditions, the spectral weights have the same general trend, but differ quantitatively in the amount of spectral weight across different shape indices l. (B) Increasing septin expression increases spectral weight at high values of l (fine texture of the surface). (C) Increasing septin expression reduces spectral weight for l=1, which is a measure of unilateral protrusion of the mesh relative to a spherical reference geometry. (D) Septin expression is correlated with greater surface roughness. Error bars represent standard error, and shaded regions in (A-B) represent standard deviations. *n* = 4 for control, 10 for knockdown, and 3 for overexpression. ∗*p* < 0.05, ∗∗*p* < 0.01, and ∗∗∗*p* < 0.001 when analyzed by one-sided t tests.

The data output includes power spectra comparisons illustrating the spectral weights of each genotype or category across each mode and bar graphs for the low-order mode comparison and high-order mode comparison. For each mesh file, the following files are generated in the ‘analysis’ output folder:

<filename>_conformalMappingToUnitSphere.mat file with information on the mapping to a spherical geometry, where <filename> is the name of each PLY file, <filename>_meanCurvature .mat file with mean curvature measurement across the surface, <filename>_powerSpectrium_radialu.mat with the spectral weight for each spherical harmonic, image files plotting the spectrum $a_{l}^{m}$ against all possible spherical harmonic indices (m,l) and showing the spectrum $A_{l}$ against indices l as histograms, comparison_of_powerSpectra_radialu.pdf in each condition’s directory (e.g., in the ‘wildtype’ folder) showing spectral weight as a function of shape index for all PLY files in the condition folder, comparison_of_powerSpectra_radialu.pdf in the parent directory (ex, in the ‘plys’ folder) showing the spectra for each condition as a shaded error plot, as in Figures 5A and 5B, statistics_radialu.mat in the parent directory with measurements of spectral weights across conditions, and comparison of spectral weight in high order modes and low order modes, as in Figures 5C and 5D, saved as both .mat files and as PDF images.

## Quantification and statistical analysis

At least three clusters should be used for each category, and a larger sample size may be required depending on the phenotype variability. We chose the categories low order mode comparison (l = 1) and high order mode comparison (l > 6) to represent the contrast between simple (mode 1) and more complex surface geometries (especially evident in modes above 6). The spectral decomposition analysis script compares experimental groups to the control with one-sided t-tests. This statistical test can clearly differentiate between groups of more or less complex geometries. Alternative statistical tests can be implemented either through direct incorporation into the MATLAB script or by exporting the raw data and using data analysis software such as GraphPad Prism.

## Limitations

This method has some limitations. If a genotype of interest results in complete detachment failure, it can be difficult to segment the border cell cluster separately from neighboring follicle cells, especially if they also express the membrane marker. It is challenging to clearly image the surface of a border cell while it is closely attached to follicle cell surfaces. In general, background signal and expression in nurse or follicle cells can affect segmentation.

There are also imaging limitations. For example, the Airyscan resolution is the limiting factor for capturing surface texture. This method will not accurately depict or compare surface texture differences at a finer scale than the imaging resolution possible. If either end of a cluster gets cut off during imaging, it is unusable for quantitative analysis. If imaging especially large border cell clusters, or samples larger than border cell clusters, it may be difficult to capture images with Airyscan due to drifting or photobleaching during the longer imaging time. We also used Airyscan 1 for this process; there may be adjustments needed to the surface texture analysis scripts if a different generation of Airyscan is used.

This current protocol is low throughput, as the surface-extraction steps to generate the 3D models are semi-automatic but not fully automatic. Measuring texture across many genotypes or samples with a range of textural variety may require a higher sample number and this process can be tedious. However, there are some ways of automating this protocol. Ilastik can batch-process multiple samples at a time after initial training, which significantly reduces the time spent training the models. Processing in MeshLab is also performed manually, but this has automation potential. We have yet to attempt this, but it may be beneficial for experiments with larger data sets. Finally, another limitation is the processing power and memory of the workstation. If the computer cannot meet the demands for the segmentation (particularly Ilastik), then this protocol will not work.

## Troubleshooting

### Problem 1

Flies are sick and/or do not survive after Gal4 activation, ovaries are small with infrequent stage 9-10 egg chambers, and/or the border cell cluster does not detach from the anterior of the egg chamber (Fly genetics and fattening for dissection step).

### Potential solution

If flies of a genotype of interest tend to be sick with small and unhealthy ovaries, increase the number of flies dissected for each sample. The expressed UAS-construct may be expressed for too long or at too high a strength that it is affecting other processes besides border cell migration. This can be mitigated by expressing the Gal4 driver for less time or by using a Gal4 driver with weaker or more specific expression. For example, if expressing an RNAi line with the Gal4 driver c306 for three days results in border cell cluster detachment failure and/or sick flies, try expressing the RNAi line for only one or two days or with an alternative border cell-specific driver such as slboGal4.

### Problem 2

The membrane marker signal is weak during imaging (airyscan image acquisition step).

### Potential solution

Ensure that the samples are not exposed to light during the fixing, washing, and immunostaining process. Samples will be briefly exposed to light during each pipetting step but they should be covered with foil while on the rocker. After mounting, the slides should be kept in the dark or covered. Fresh samples mounted within the last day or two will have the strongest signal, and the signal will be lost over time. If samples have been prepared but there is no time to image them, they will preserve more of their signal left in refrigerated Vectashield rather than mounted on slides. Membrane markers in the GFP channel tend to have the best signal, so select a marker in the GFP channel, if possible. If using an antibody or dye, try increasing its concentration. During imaging set-up, increase the gain and/or laser power to pick up more signal.

### Problem 3

The sample drifts during imaging (airyscan image acquisition step).

### Potential solution

To avoid drifting problems, ensure the microscope is seated on a functional air table that resists shaking. During the mounting process, do not use more than 60–70 μL of Vectashield. Avoid incorporating any air bubbles into the sample. The microscope lasers can slowly melt away the nail polish sealant on the coverslip edges, so make sure to generously coat the coverslip edges with 1–2 layers of nail polish. The day before, or at least a few hours before imaging, allow the slide to sit at 25°C (covered or in the dark). If the slide is too cold, the coverslip can warp and the Vectashield can shift during imaging. In addition, decreasing the microscope stage speed in Zen can reduce drifting.

### Problem 4

Errors from not having packages installed or from not initializing setup.m before running the STAR_Methods_Tissue_Cartography_Modeling.m script (extract the surface of the cluster using ImSAnE step).

### Potential solution

Double-check that all toolboxes listed in Step: Software and package installation and set-up have been properly installed. Run setup.m from the ImSAnE repository. Try restarting MATLAB after making these changes to ensure that MATLAB has properly initialized with the toolboxes.

### Problem 5

Errors from not having the directory properly organized or misnamed files (extract the surface of the cluster using ImSAnE step).

### Potential solution

Verify that all ImSAnE files are in the same directory and that MATLAB can access those files. Make sure that MATLAB is running in the correct directory. This can be verified by typing ‘pwd’ in the command window and pressing “enter”, which will then show the current working directory. Double-check the file names throughout the script to ensure they match the file names in the directory.

### Problem 6

The point cloud model contains extraneous areas or a secondary object that is not the object of interest (extract the surface of the cluster using ImSAnE step).

### Potential solution

Additional training in Ilastik is required to further exclude these pixels.

### Problem 7

Errors from over- or under-sampling for Poisson Disk Sampling in MeshLab (generate 3D curvature models step).

### Potential solution

If the MATLAB script displays errors after using MeshLab, try running MeshLab again and increase the sample number to 10,000. If the error persists, try using a sample number of 5,000. If neither of these solutions works, delete the MeshLab file, re-import the PLY file, and start the MeshLab section of the protocol again.

### Problem 8

Cluster is cut off in the final 3D rendering (generate 3D curvature models step).

### Potential solution

The image can get cut off at multiple points in this process. Start by scrolling through the z-stack images and ensuring that the original image is not truncated at the top or bottom. This is the most likely issue, and, unfortunately, means that the image is not usable. If the images look good at that step, then re-train the Ilastik model and take extra care at the top and bottom of the cluster. This should resolve the issue, so you can run the MATLAB script again with the new probabilities file from Ilastik. If Ilastik training does not solve the issue and the point cloud appears as a complete cluster, then try using a higher sample number in MeshLab to ensure the cluster has sufficient sampling.

### Problem 9

MATLAB returns an error during the script_spectralAnalysis_basic.m script saying that the conformalized mean curvature flow fails to converge (spectral decomposition of border cell cluster shape step).

### Potential solution

This is probably because the mesh triangulation has triangles with large differences in their interior angles. Go back to the MeshLab steps and run an LS3 Loop with a smaller target triangle size, or use MATLAB to remesh the surface so that triangles are generally closer to being equilateral. Related warnings may appear in these cases, such as.

> Warning: Matrix is singular to working precision.

> > In conformalized_mean_curvature_flow

In script_spectralAnalysis

## Resource availability

### Lead contact

Further information and requests for resources and reagents should be directed to and will be fulfilled by the lead contact, Denise Montell (dmontell@ucsb.edu).

### Technical contact

Technical questions should be directed to the technical contact, Allison Gabbert (amgabbert@ucsb.edu).

### Materials availability

Drosophila lines and other reagents generated in this study will be available upon request.

### Data and code availability

Data including all raw image files in this study will be made available upon request. All original code has been deposited in the GitHub repository AllisonGabbert/SeptinManuscriptData and archived in Zenodo (https://doi.org/10.5281/zenodo.10791062). Any additional information required to reanalyze the data reported in this paper is available from the lead contact upon request.
